# Shell Layer Thickness-Dependent Photocatalytic Activity of Sputtering Synthesized Hexagonally Structured ZnO-ZnS Composite Nanorods

**DOI:** 10.3390/ma11010087

**Published:** 2018-01-07

**Authors:** Yuan-Chang Liang, Ya-Ru Lo, Chein-Chung Wang, Nian-Cih Xu

**Affiliations:** Institute of Materials Engineering, National Taiwan Ocean University, Keelung 20224, Taiwan; yalulo0807@gmail.com (Y.-R.L.); abc2589tw@gmail.com (C.-C.W.); sad821008@gmail.com (N.-C.X.)

**Keywords:** sputtering, composite nanorods, shell thickness, photocatalytic activity

## Abstract

ZnO-ZnS core-shell nanorods are synthesized by combining the hydrothermal method and vacuum sputtering. The core-shell nanorods with variable ZnS shell thickness (7–46 nm) are synthesized by varying ZnS sputtering duration. Structural analyses demonstrated that the as-grown ZnS shell layers are well crystallized with preferring growth direction of ZnS (002). The sputtering-assisted synthesized ZnO-ZnS core-shell nanorods are in a wurtzite structure. Moreover, photoluminance spectral analysis indicated that the introduction of a ZnS shell layer improved the photoexcited electron and hole separation efficiency of the ZnO nanorods. A strong correlation between effective charge separation and the shell thickness aids the photocatalytic behavior of the nanorods and improves their photoresponsive nature. The results of comparative degradation efficiency toward methylene blue showed that the ZnO-ZnS nanorods with the shell thickness of approximately 17 nm have the highest photocatalytic performance than the ZnO-ZnS nanorods with other shell layer thicknesses. The highly reusable catalytic efficiency and superior photocatalytic performance of the ZnO-ZnS nanorods with 17 nm-thick ZnS shell layer supports their potential for environmental applications.

## 1. Introduction

The high electron mobility, wide and direct band gap (3.1 eV–3.4 eV) and large exciton binding energy (60 meV) at room temperature spread various potential applications of ZnO [1]. Moreover, ZnO is also a cost-efficient and environment-friendly material; therefore, it is promising for applications to alleviate environmental problems. Photoexcited electron–hole pairs in ZnO under light irradiation can interact with the O_2_ adsorbed on the surface of the ZnO photocatalyst and H_2_O to generate O_2_^−^ and ·OH, respectively, which can reduce and oxidize the organic contaminants; the ZnO is promising for photocatalytic applications [2]. Changing the morphology of ZnO is an efficient way to enhance its photocatalytic efficiency. Various works have reported low-dimensional ZnO with different morphologies in photocatalyst applications [3,4]. In these studies, ZnO nanorods are posited to be a suitable architecture for photocatalytic applications [5]; moreover, ZnO nanorods coupled with other semiconductors to form core–shell nanostructures are a viable strategy to realize the efficient separation of photoinduced charge carriers in order to improve the photocatalytic performance of the ZnO nanorods. The TiO_2_-coated ZnO nanorods, CdS-coated ZnO nanorods, and Sn_2_S_3_-coated ZnO nanorods are reported to show superior photocatalytic performance than that of pure ZnO nanorods [6,7,8]. In addition, ZnS is a wide bandgap semiconductor (3.6 eV) [9]. ZnS can be designed in many forms, like particles, thin films, wires, rods, tubes, and sheets [10]. Recent work shows that ZnS is also a promising photocatalyst [11]. It has been shown that the formation of hollow ZnO core-ZnS shell structure improves the photocatalytic activity of pristine ZnO [12]. Moreover, when compared with ZnO spheres, the photocatalytic activities for methyl orange of the ZnO-ZnS core-shell sphere structure is improved [13]. Theoretical calculations and experimental results have demonstrated that the combination of the ZnO and ZnS wide bandgap semiconductors can yield a novel material that has photo-induced threshold energy lower than that of the individual components [14]. Based on the aforementioned discussions, the integration of ZnS into ZnO nanorods is a promising approach to enhance the photocatalytic properties of the ZnO nanorods.

Covering ZnS shell layer crystallites onto the ZnO nanorods, a proper thin-film process of ZnS is crucial to fabricate ZnO-ZnS core-shell composite nanorods with a successive architecture. Several techniques, including sputtering [15], thermal evaporation [16,17], pulsed laser deposition, [18], and chemical bath deposition [19] have been used to grow ZnS thin films. In particular, the sputtering technique is the most promising method for up-scaling the growth system while maintaining a good control of the deposition rate, so it is widely used in industrial applications [20,21]. In the present study, ZnS crystallites with various thicknesses were sputtered onto ZnO nanorods to form ZnO-ZnS core-shell heterostructured nanorods. The correlation between microstructures and photocatalytic properties of the sputtering formation of ZnO-ZnS nanorods was investigated.

## 2. Materials and Methods

In this study, ZnO-ZnS core-shell nanorods with different ZnS shell layer thicknesses were fabricated by sputtering ZnS thin films with different sputtering durations onto the surfaces of hydrothermally derived ZnO nanorod templates. The synthesis of the ZnO nanorods consisted of two steps corresponding to the formation of a ZnO seed layer and the growth of nanorods. In the first, the ZnO seed layer was deposited on the 300 nm-thick SiO_2_/Si substrate by magnetron sputtering. Subsequently, the substrates were perpendicularly suspended in a solution containing equimolar (0.05 M) aqueous solutions of zinc nitratehexahydrate (Zn(NO_3_)_2_∙6H_2_O) and hexamethylenetetramine (C_6_H_12_N_4_). The hydrothermal reaction temperature was fixed at 95 °C and the duration for crystal growth is 9 h. The as-synthesized ZnO nanorods have the average diameter of 135 nm. Moreover, the average length of the ZnO nanorods is 1.36 µm. The ZnS shell layer was deposited on the ZnO nanorods by radio-frequency (RF) magnetron sputtering of a ZnS target. ZnS film was deposited in pure Ar ambient at 80 W under 2.67 Pa working pressure. The ZnS film growth temperature was fixed at 460 °C. The thickness of the ZnS shell was varied by controlling the sputtering duration from 10 to 60 min.

Sample crystal structures of the ZnO-ZnS composite nanorods were investigated by X-ray diffraction (XRD, Bruker D2 PHASER, Karlsruhe, Germany) using Cu Ka radiation. The surface morphology of the composite nanorods was characterized by scanning electron microscopy (SEM, Hitachi S-4800, Tokyo, Japan). The crystallinity and thickness of the ZnS shell layer were measured by transmission electron microscopy (TEM, Philips Tecnai F20 G2, Amsterdam, The Netherland). The rod-like thin film samples with a thickness of approximately 1.36 µm are used for the room-temperature photoluminescence (PL) measurements. The Horiba Jobin Yvon HR 800 (Horiba Scientific, Kyoto, Japan) with the 325 nm continuous wave He-Cd laser source are used in the emission measurements. To measure photocurrent properties of the composite nanorods, solarlight irradiation excited from a 100 W Xe arc lamp was used for illumination. Silver glues were laid on the surfaces of the samples to form two contact electrodes, and the applied voltage was fixed at 5 V during electric measurements. Photocatalytic activity of various ZnO-ZnS composite nanorods were performed by comparing the degradation of aqueous solution of methylene blue (MB, 5 × 10^−6^ M) containing various ZnO-ZnS nanorods as catalysts under solarlight irradiation excited from a 100 W Xe arc lamp. The solution volume of MB is 10 mL and the ZnO-ZnS nanorods are grown on the substrates with a fixed coverage area of 1.2 cm × 1.2 cm for the photodegradation tests. The variation of MB solution concentration in the presence of various ZnO-ZnS nanorods with different irradiation durations was analyzed by recording the absorbance spectra using an UV-Vis spectrophotometer (Jasco V750, Tokyo, Japan).

## 3. Results and Discussion

**Figure 1:**
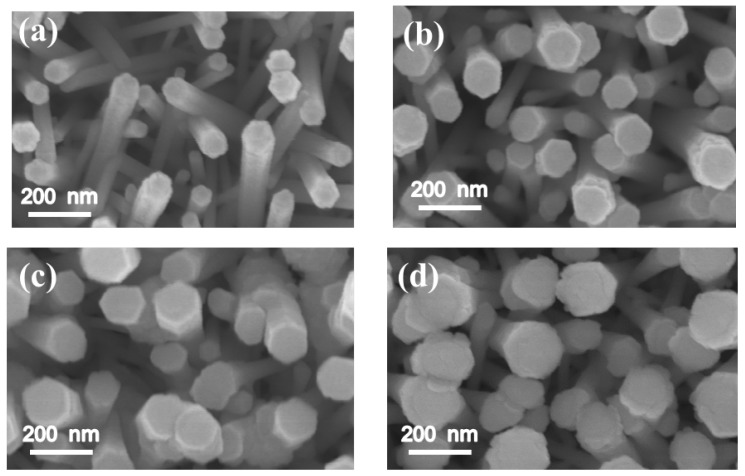
SEM images of ZnO-ZnS core-shell nanorods with various ZnS sputtering durations: (**a**) 10 min; (**b**) 20 min; (**c**) 40 min and (**d**) 60 min.

**Figure 2:**
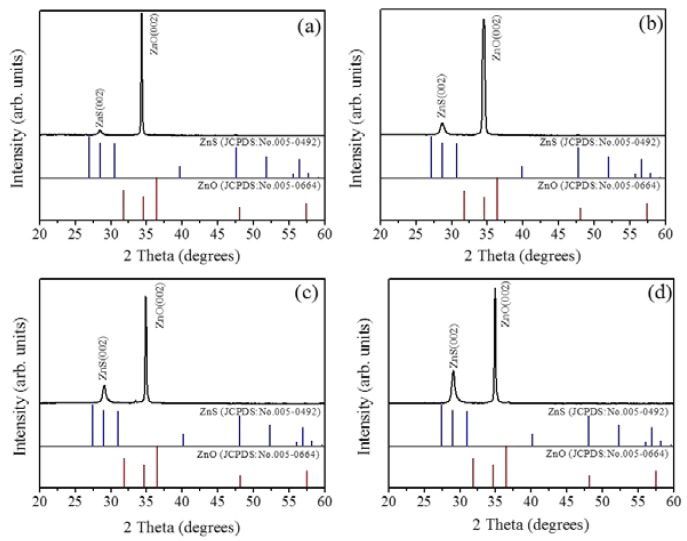
X-ray diffraction (XRD) patterns of ZnO-ZnS core-shell nanorods with various ZnS sputtering durations: (**a**) 10 min; (**b**) 20 min; (**c**) 40 min and (**d**) 60 min.

Figure 1a–d show the SEM images of the as-synthesized ZnO-ZnS core–shell nanorods with various ZnS sputtering durations. Upon increasing the sputtering duration, a marked increase in the diameter of the composite nanorods was visible. In the heterostructure systems of sputtering deposited perovskite La_0.7_Sr_0.3_MnO_3_ epilayers on SrTiO_3_ substrates and (La,Ba)MnO_3_ films on SrTiO_3_ substrates, a thicker film engenders roughening of surface morphology [22,23]. The lattice misfit between the as-grown film and the underlayer material causes the marked thickness-dependent roughness scaling effect. The ZnS and ZnO have the same wurtize hexagonal structure, but different lattice constants. An increase in sputtering deposited ZnS shell layer thickness might cause increased layer surface undulation. Figure 2a–d exhibit XRD patterns of the ZnO-ZnS composite nanorods with various ZnS shell layer thicknesses. The intense Bragg reflection that was centered at approximately 34.4° is ascribed to the ZnO (002). No other Bragg reflection from the ZnO was distinguished revealed that the highly c-axis oriented feature of the ZnO nanorods. In addition to ZnO (002), an obvious Bragg reflection centered at approximately at 28.6° is noticed which corresponds to (002) plane of wurtzite ZnS (JCPDS No. 005-0492). The relative intensity of the ZnS (002) Bragg reflection increased upon increasing the ZnS sputtering duration and no other Bragg reflection originated from other crystallographic planes was observed. This indicates that hexagonal ZnO crystal provides well crystallographic accommodation for the growth of hexagonal ZnS shell layers by sputtering deposition at 460 °C in this study [24]. By contrast, the zinc blend cubic ZnS phase is favorable to form at a low-temperature sputtering process and the substrate type affects the crystallographic structure of ZnS [25]. It has been shown that the ZnO-ZnS composites with various morphologies composed of hexagonal ZnO and cubic ZnS are formed at other low temperature synthesis methods [12]. The sputtering growth of the ZnS shell layer herein provides ZnS adatoms with sufficient energy to adsorb onto the ZnO facets and growth with the similar crystallographic feature of the under-layered ZnO crystal at the given growth temperature [26]; therefore, the ZnO-ZnS composite nanorods with single hexagonal phase were formed.

**Figure 3:**
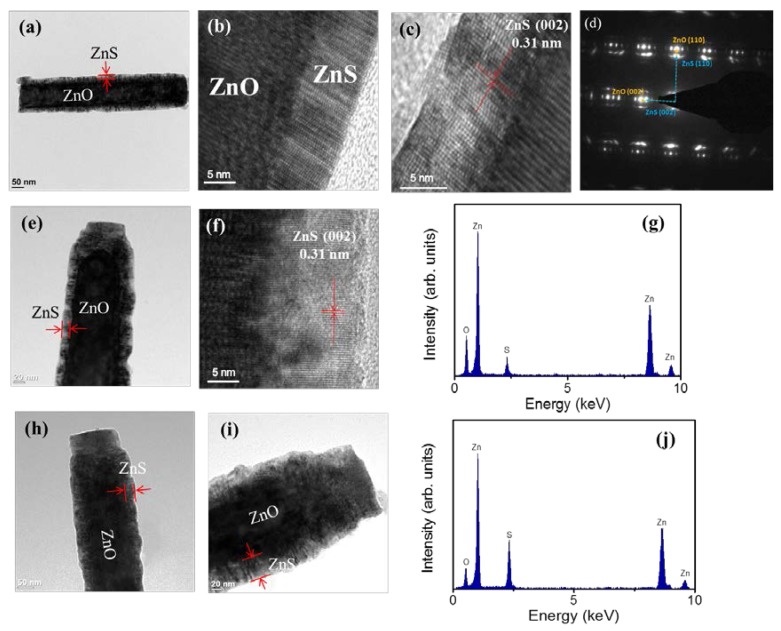
(**a**) Low-magnification transmission electron microscopy (TEM) imageof ZnO-ZnS composite nanorod with ZnS sputtering 10 min; the thickness of ZnS is approximately 7 nm; (**b**,**c**) The corresponding high-resolution images obtained from the various regions of the composite nanorod; (**d**) Selected area electron diffraction (SAED) pattern of the nanorod; (**e**) Low-magnification TEM imageof ZnO-ZnS composite nanorod with ZnS sputtering 20 min, the thickness of ZnS is approximately 17 nm; (**f**) The corresponding high-resolution image obtained from the outer region of the nanorod; (**g**) Energy dispersive spectroscopy (EDS) spectra of the nanorod; (**h**,**i**) Low-magnification TEM images of ZnO-ZnS composite nanorods with ZnS sputtering 40 min and 60 min; the thicknesses of ZnS are approximately 32 nm and 46 nm, respectively. (**j**) EDS spectra of the ZnO-ZnS nanorod in (**i**).

Figure 3a shows a low-magnification TEM image of a single ZnO-ZnS core–shell nanorod with the ZnS sputtering duration of 10 min. An ultrathin ZnS layer was homogeneously covered over the surface of the ZnO nanorod. The thickness of the ZnS shell layer is estimated to be approximately 7 nm. Figure 3b,c show the high-resolution TEM (HRTEM) images of the ZnO-ZnS core–shell nanorod taken from the different local regions at the ZnO-ZnS interface. The arrangement of lattice fringes of the shell layer is visible and highly ordered and correlated with those of the ZnO core, revealing that the atomic arrangement orientation of the ZnO and ZnS crystals is in a similar manner. The clear lattice fringes in the HRTEM images with an inter-planar spacing of approximately 0.31 nm corresponded to the (002) plane of the hexagonal ZnS structure. The arrangement of lattice fringes in Figure 3b,c demonstrates the crystal orientations of the ZnO and ZnS match well along (002) plane. Figure 3d exhibits selected area electron diffraction (SAED) pattern of the ZnO-ZnS nanorod. The SAED pattern shows that both ZnO core and ZnS shell crystals are grown along c-axis direction. Because of the similar crystal structure and the crystallographic growth orientation between the constituent compounds, the satellite spots are visible. This is associated from the reconstruction of the diffraction from the overlapping planes of the ZnO and ZnS [27]. The TEM analyses revealed that the ZnO-ZnS nanorod synthesized by sputtering ZnS shell layer has a high crystallinity. Figure 3e displays low-magnification TEM image of the ZnO-ZnS nanorod with 20 min sputtering growth of ZnS shell layer; the as-deposited ZnS shell thickness is estimated approximately 17 nm. Moreover, Figure 3f demonstrates that the ZnS shell layer still exhibited well-ordered arranged lattice fringes, revealing the highly crystallinity of the ZnS crystallites. The energy dispersive spectroscopy (EDS) spectra in Figure 3g shows that Zn, O, and S are the primary detected elements and no other impurity atoms are detected. Figure 3h,i display the low-magnification TEM images of the ZnO-ZnS nanorods with 40 min and 60 min ZnS sputtering durations; the outer gray-layer is originated from the ZnS. The thicknesses of the ZnS shell layer were estimated to around 32 nm and 46 nm, respectively. The EDS spectra of the ZnO-ZnS nanorod with a 46 nm-thick ZnS shell layer were also displayed in Figure 3j. Notably, since the ZnS shell layer thickness was thickened by a prolonged sputtering process, the intensity of sulfur signal was markedly increased compared with that shown in Figure 3g wherein the core-shell composite nanorod has a thinner ZnS shell layer.

**Figure 4:**
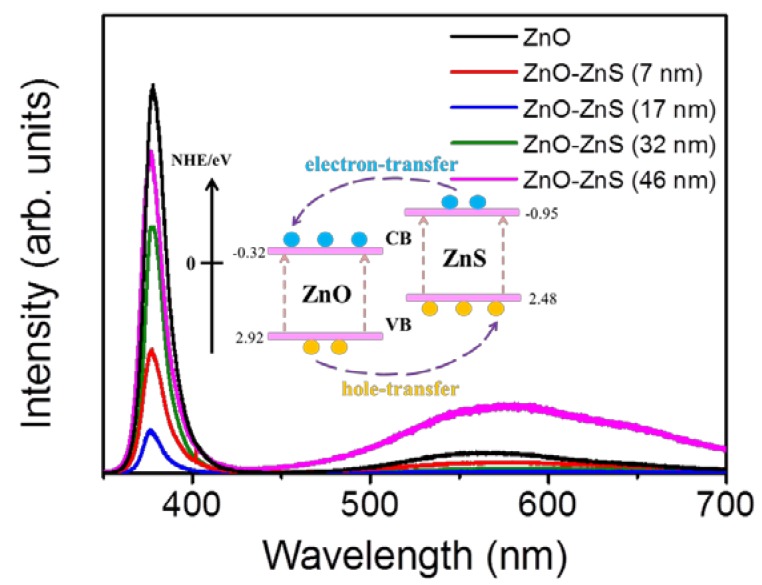
PL spectra of ZnO-ZnS composite nanorods with various sputtering ZnS thicknesses.

Figure 4 illustrates the PL spectra of the ZnO-ZnS core–shell nanorods with different ZnS shell layer thicknesses. A sharp and distinct UV emission band was ascribed to the near-band edge (NBE) emission of the ZnO nanorods [8]. Moreover, a broad and clear visible-light emission band centered at approximately 570 nm was observed for the ZnO nanorods. This green-yellow band for visible luminescence is due to the discrete deep energy levels in the band gap formed by the point defects [28]. Notably, the NBE emission intensity of the ZnO nanorods was quenched when the ZnS shell layer was coated onto the surfaces of the ZnO nanorods. Moreover, the ZnO-ZnS nanorods with a 17 nm-thick ZnS shell layer exhibited a substantial drop in NBE intensity when compared with other ZnO-ZnS nanorods. Coating the surfaces of the ZnO nanorods with ZnS shell layer leads to fill up the oxygen vacancies on the surface of the oxide nanorods with the S atoms. The intensity ratio of visible emission band peak to NBE peak (I_D_/I_NBE_) for the ZnO nanorods was 8.4%. The decoration of ZnS shell layer (7 nm) onto the surfaces of the ZnO nanorods decreased the NBE peak intensity and the I_D_/I_NBE_ ratio was also decreased to 3.8%. Further increasing the ZnS shell layer thickness, more S atoms fill up more oxygen vacancies with an increased ZnS shell layer thickness and further decreases the I_D_/I_NBE_ ratio to 2.1% and 1.8% for the ZnO-ZnS (17 nm) and ZnO-ZnS (32 nm), respectively. However, further thickening the ZnS shell layer to 46 nm, the position of deep level emission band of the ZnO-ZnS nanorods was markedly red-shifted and exhibited a substantial increase in intensity (I_D_/I_NBE_ = 14.8%). A lot of surface oxygen vacancies of the ZnO nanorods have been filled up with an increased ZnS shell layer and this engendered a decreased deep level emission of the ZnO nanorods. The formation of a thicker ZnS layer (46 nm), in contrast, might form more sulfur vacancies in the ZnS crystallites with the prolonged sputtering duration, which resulted in the presence of visible deep level emission that originated from the ZnS shell crystallites [29]. Comparatively, an optimal ZnS shell layer thickness of approximately 17 nm was found to make ZnO-ZnS nanorods showing superior photoexcited charge separation efficiency between the ZnO-ZnS heterointerface.

**Figure 5:**
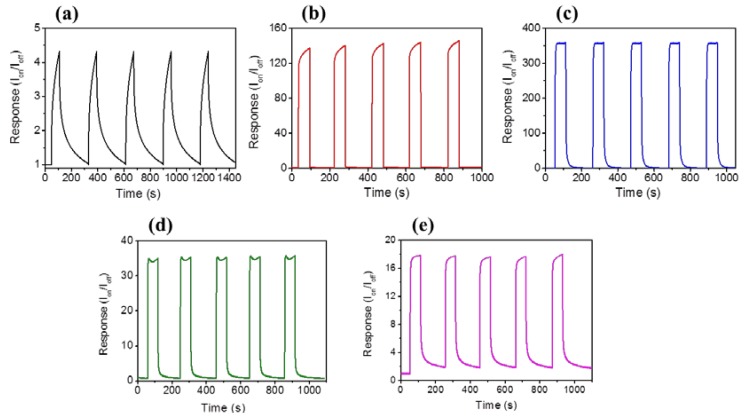
Photoresponse curves of various nanorods: (**a**) Pure ZnO; (**b**)ZnO-ZnS (7 nm); (**c**) ZnO-ZnS (17 nm); (**d**)ZnO-ZnS (32 nm); (**e**) ZnO-ZnS (46 nm).

Figure 5a–e display photoresponse behaviors of the ZnO nanorods and various ZnO-ZnS core-shell nanorods. The measured photocurrents increased as a function of time after the sample was exposed to solar light, and successfully reached the steady-state value. The measured photocurrents of the sample decreased over time in the absence of solar light. The ratio of solar light irradiated currents (I_on_) to dark currents (I_off_) is used to determine the photoresponse performance of the composite nanorods [30]. Notably, the photoresponse of the ZnO nanorods is approximately 4.4. A substantial enhancement of the photoresponse of the ZnO nanorods was observed for the surface decoration with a ZnS shell layer. The conduction band energy level of ZnS is found to be energetically slightly higher than that of ZnO [31,32]. A possible band alignment between the ZnO and ZnS was shown in the inset of Figure 4 [33]. When the ZnO-ZnS nanorods were exposed to solar light, a large number of photoexcited electron-hole pairs are formed in both ZnS and ZnO. The electrons are readily from the conduction band of ZnS to the conduction band of ZnO. The well crystalline ZnO nanorods provide a path for transporting electrons to the adjacent electrodes and an enhanced photoresponse was observed for the ZnO nanorods coated with the ZnS shell layer. Similar photoresponse enhancement mechanism has been reported in ZnO-Cu_2_O and ZnS-SnO_2_ heterostructure systems, wherein a similar band alignment structure exists between the counterparts [34,35]. The photoresponse of the ZnO-ZnS nanorods with the 7 nm-thick ZnS shell layer is approximately 138. An increase of the ZnS shell layer thickness to 17 nm makes the ZnO-ZnS nanorods exhibit the highest photoresponse of approximately 358. However, further thickening the ZnS shell layer to 32 nm and 46 nm, by contrast, decreased the photoresponses of the ZnO-ZnS composite nanorods to 34 and 18, respectively. The photoresponse of nanorods is largely affected by the number of surface adsorbed O_2_ and H_2_O molecules [36]. The shell layer thickness has been shown to affect photocurrent size of the heterostructure systems. It has been shown in the ZnO-TiO_2_ composite nanorods with various TiO_2_ shell layer thicknesses, an increased coating cycle number of TiO_2_ shell layer engenders the photoexcited electrons produced in the shell are trapped by the adsorbed oxygen molecules on the surface of TiO_2_, and thus could not be transferred to ZnO. This results in a large drop in the photocurrent in the ZnO-TiO_2_ system [37]. In addition, the ZnO-ZnS core-shell nanorods herein also display shorter response time and recovery time than those of pure ZnO nanorods. The response time was 68 s and the recovery time was 108 s for the ZnO nanorods. However, the response time and recovery time of the ZnO-ZnS (17 nm) was markedly shortened to 30 s and 78 s, respectively. This is associated with the charges combined with type-II band structure between ZnO and ZnS, which can easily separate the photoexcited electrons and holes into the ZnO core and ZnS shell, respectively. Notably, the PL analysis result revealed that the charge separation efficiency of the ZnO-ZnS was substantially improved with the 17 nm-thick ZnS shell layer; this is correlated with the highest photoresponse of the ZnO-ZnS (17 nm) herein.

**Figure 6:**
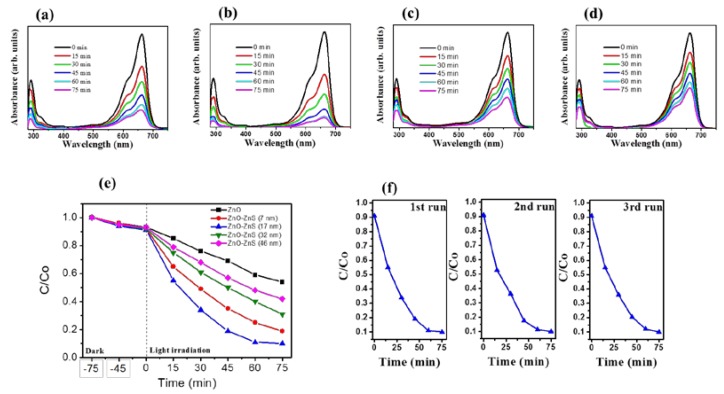
Intensity variation of absorbance spectra of MB solution vs. degradation duration containing various ZnO-ZnS nanorods with different sputtering ZnS thicknesses: (**a**) 7 nm; (**b**) 17 nm; (**c**) 32 nm; (**d**) 46 nm; (**e**) C/C_o_ vs. irradiation time for MB solution containing various ZnO-ZnS nanorods in dark conditions and under solarlight illumination. For comparison those of pure ZnO nanorods are also shown in the plot; (**f**) Recycled photodegradation performances (three test runs) of MB solution containing the ZnO-ZnS (17 nm) nanorods.

The photocatalytic performance of ZnO-ZnS nanorods with various ZnS shell layers under light irradiation was further investigated. The time-course absorbance spectra for an aqueous MB solution after photodegradation containing various ZnO-ZnS nanorod samples are displayed in Figure 6a–d. The visible and intense peaks of the absorbance spectra at approximately 663 nm were due to the monomeric MB; the intensity of absorbance spectra decreased with irradiation time. This result indicates that MB dyes are gradually degraded under irradiation. The ratio of the remaining MB concentration (C) after light irradiation to the initial MB concentration without light irradiation (C_o_) i.e., C/C_o_ was further used to determine the photodegradation level of the MB solution containing various nanorod samples. The C/C_o_ value was evaluated from the absorbance spectra intensity ratio at 663 nm before and after the MB solution was subjected to irradiation with various nanorod samples. The C/C_o_ versus irradiation duration results for the MB solution containing various nanorod samples are shown in Figure 6e. Notably, dark adsorption tests were performed for various durations before the photocatalytic degradation tests under irradiation. The MB concentration changes in dark conditions are influenced by the adsorption of MB dyes on the surfaces of various nanorod samples. Notably, photoexcited electrons or holes in the ZnO-ZnS nanorods were transferred to the active surface and join in the redox reactions. The producing hydroxyl radicals were strong oxidizing agents and effectively decomposed the MB dyes [38]. Under the given irradiation duration, the complex ZnO-ZnS (17 nm) nanorods show much greater photocatalytic activity in the decomposition of MB when compared to other ZnO-ZnS nanorods. Almost 90% of the MB solution containing ZnO-ZnS (17 nm) nanorods was discolored under irradiation for 60 min. However, only 75%, 60%, 52%, and 40% MB solution were discolored containing ZnO-ZnS (7 nm), ZnO-ZnS (32 nm), ZnO-ZnS (46 nm), and ZnO nanorods, respectively, under irradiation for 60 min. Further extended the irradiation duration to 75 min, the discoloration level of MB solution containing ZnO-ZnS (17 nm) reached a balance value of 90%, revealing that most MB dyes in the solution were degraded in the presence of the ZnO-ZnS (17 nm) nanorods under irradiation after 60 min. By contrast, the discoloration level of the MB solution containing other nanorods was further increased and did not reach a balance value. The complex ZnO-ZnS (17 nm) nanorods exhibited the suitable shell thickness of ZnS to enhance electron–hole separations at the interface of ZnO-ZnS, resulting in the highest photocatalytic efficiency. The similar improvement in photocatalytic efficiency and stability of the SnO_2_ has been achieved in coupling SnO_2_ with ZnS to form a heterostructure. The efficient separation of hole–electron pairs at the SnO_2_-ZnS heterointerface has posited to suppress photoexcited charge recombination and inhibit photocorrosion of SnO_2_-ZnS nanocomposites [39]. The PL results and photoresponse performance of the ZnO-ZnS (17 nm) nanorods supported the superior photocatalytic activity of the ZnO-ZnS (17 nm) nanorods, herein. Notably, the substantial decrease in the absorbance peak intensity in the UV region is associated with the mineralization of the MB dyes during the photodegradation process [7]. The trend that the larger intensity decrease level of the absorbance peak in the UV region for the MB solution containing ZnO-ZnS (17 nm) nanorods at the given irradiation duration is in accordance with the observations on discoloration degree of the MB solution containing various nanorod samples. The stability and reusability of the ZnO-ZnS (17 nm) nanorods were evaluated by performing recycling reactions three times for the photodegradation of the MB solution under irradiation. In Figure 6f, after three cycles, the ZnO-ZnS (17 nm) nanorods maintain the high reusability and stability; an approximately 90% photodegragation of the MB solution was observed. The ZnO-ZnS (17 nm) nanorods are advantageous in the applications of photocatalyst for organically polluted water treatment.

## 4. Conclusions

We have effectively demonstrated the photocatalytic properties of shell thickness controlled ZnO-ZnS core-shell nanorods, formed through sputtering ZnS thin films with different sputtering durations onto the surfaces of ZnO nanorod templates. The crystal structure analyses revealed that the shell layer is well crystallized, with preferring growth direction of ZnS (002). This indicates that hexagonal ZnO crystal provides well crystallographic accommodation for growth of hexagonal ZnS shell layers by sputtering deposition at 460 °C, herein. Among various ZnS shell layer thicknesses, the ZnO-ZnS nanorods with the 17 nm-thick ZnS shell layer exhibited the highest photoexcited charge separation efficiency; therefore, displayed the highest photodegradation level to MB dyes. Moreover, the ZnO-ZnS (17 nm) nanorods maintained the high reusability and stability after three cycles degradation. The results herein imply that the control of sputtering deposited ZnS shell layer thickness and crystallinity is an efficient approach to optimize the charge separation efficiency of ZnO-ZnS core-shell nanorods, and, therefore, improving photocatalytic activity of the composite nanorods.
